# Differences in the relationship of weight to height, and thus the meaning of BMI, according to age, sex, and birth year cohort

**DOI:** 10.1080/03014460.2020.1737731

**Published:** 2020-05-20

**Authors:** William Johnson, Tom Norris, David Bann, Noël Cameron, Jonathan K. Wells, Tim J. Cole, Rebecca Hardy

**Affiliations:** a School of Sport, Exercise and Health Sciences, Loughborough University, Loughborough, UK;; b Centre for Longitudinal Studies, UCL Institute of Education, London, UK;; c UCL Great Ormond Street Institute of Child Health, London, UK;; d CLOSER, UCL Institute of Education, University College London, London, UK

**Keywords:** Benn parameter, Body mass index, birth year cohort, secular trend, trajectory

## Abstract

**Background:** Weight can be adjusted for height using the Benn parameter (kg/m^B^), where B is the power that minimises the correlation with height.

**Aim:** To investigate how the Benn parameter changes across age (10–65 years) and time (1956–2015) and differs between sexes.

**Subjects and methods:** The sample comprised 49,717 individuals born in 1946, 1958, 1970 or 2001. Cross-sectional estimates of the Benn parameter were produced and cohort differences at ages 10/11 and 42/43 years were examined using linear regression. Multilevel modelling was used to develop trajectories showing how the Benn parameter changed over age from childhood to mid-adulthood in the three older cohorts.

**Results:** The Benn parameter was closest to 2 in childhood but consistently lower across adulthood, particularly in females and the most recent cohort. At ages 10/11 years, the Benn parameter was greater than 3 in both sexes in the 2001 cohort but between 2.2 and 2.7 in the three older cohorts. This difference was estimated to be +0.67 (0.53, 0.81) in males and +0.53 (0.38, 0.68) in females, compared to the 1946 cohort, and was driven by a much higher weight SD in the 2001 cohort. Conversely, at ages 42/43 years, the Benn parameter was lowest in the 1970 cohort due to a slightly lower weight-height correlation. This difference was estimated to be −0.12 (−0.34, 0.10) in males and −0.15 (−0.42, 0.13) in females, compared to the 1946 cohort.

**Conclusions:** Changes over time in the obesogenic environment appear to have firstly reduced the Benn parameter due to a lowering of the weight-height correlation but secondly and more drastically increased the Benn parameter due to increasing weight variation.

## Introduction

The body mass index (BMI kg/m^2^) is an index of weight-for-height that works on the premise that weight increases proportionately to height squared, so that dividing weight by height squared results in an index that is uncorrelated with height. This is an attractive quality for researchers and clinicians who want to analyse or assess body weight while accounting for the fact that taller people are generally heavier and shorter people are generally lighter. The index was initially called the Quetelet Index after the Belgian scientist Lambert Adolphe Quetelet (Quetelet 1842). Over the following 100 or so years, numerous other formulae were proposed, including the Rohrer index (kg/m^3^) (Rohrer 1908; Keys et al. 1972). In a review of the published literature, Ancel Keys et al (1972) set out to decide which was the best index based on (1) the strongest correlation with weight and adiposity and (2) the weakest correlation with height. Quetelet’s Index was found to be the best “obesity index” and a change in name to the BMI was proposed. Keys’ work was, however, firmly aimed at adults. In the 1970s, weight-for-height was used in children without reference to age, partly due to convenience and also because no-one recognised the need to adjust for age as well as height. Cole’s (1979) seminal paper was the first to propose that BMI adjusted for age was a useful alternative to weight-for-height in children. By the 1980s, the BMI was used by many human biologists to adjust weight for height in both children and adults (Deutsch et al. 1985; Rosenbaum et al. 1985; Lasker and Mascie-Taylor 1989).

Today, the BMI is ubiquitous in obesity research and practice but is also widely criticised (Prentice and Jebb 2001) mainly for showing residual correlation with height and failing to distinguish between fat mass and fat-free mass (Romero-Corral et al. 2008), particularly in early-childhood (Demerath et al. 2006; Johnson et al. 2017). Note that solving this problem by measuring body composition simply transmits the dilemma of what powers of height to use to each of fat mass and fat-free mass (Wells et al. 2002; Nightingale et al. 2011). The BMI was never intended to assess obesity status in an individual but might be improved in any given sample by altering the height power to make the index truly uncorrelated with height. The Benn index does exactly this and can be calculated as kg/m^B^, where the Benn parameter (B) is the coefficient obtained from a general linear regression of log weight (kg) on log height (m). A coefficient of 2, as used in the calculation of BMI, would mean that as height increases by 1% weight increases by 2%. The coefficient can, of course, also be calculated as

$$B=r\frac{S_{y}}{S_{X}}$$

where r is the correlation between log weight and log height, $S_{y}$ is the standard deviation (SD) of log weight, and $S_{X}$ is the SD of log height (Stanton 2001).

When expressed this way, it is easy to see how the Benn parameter is influenced by variation in, and the correlation between, weight and height. Previous studies have investigated the value of the Benn parameter in different samples and at different ages, generally finding that values peak at 2.5–3.0 in puberty before declining into adulthood, where values are normally between 1.0 and 2.5 (Goldbourt and Medalie 1974; Lee et al. 1981; Garn and Pesick 1982; Cole 1986; Micozzi et al. 1986; Revicki and Israel 1986; Nevill and Holder 1995; Sperrin et al. 2016; Peterson et al. 2017). The peak occurs when it does because puberty leads to greater variation in weight and height, which stretches the weight–height ellipse along its major axis, increasing the weight–height correlation and therefore the Benn parameter. Despite there being a long history of research on this topic, no studies have modelled longitudinal data to produce trajectories showing how the Benn parameter changes with age from childhood to mid-adulthood. Previous studies have also consistently reported the Benn parameter to be lower in females than males (Goldbourt and Medalie 1974; Lee et al. 1981; Garn and Pesick 1982; Cole 1986; Micozzi et al. 1986; Revicki and Israel 1986; Nevill and Holder 1995; Sperrin et al. 2016; Peterson et al. 2017). This difference, at least in adulthood, has been explained by Burton as resulting from the fact that women have greater percentage body fat than men (Burton 2007; Flegal et al. 2009), which lowers the value of the Benn parameter because fat mass is highly variable yet has a very low correlation with height (Heymsfield et al. 2007; Burton 2015). Put differently, the greater the level of adiposity, the lower the correlation of total body weight with height (because fat mass has a very low correlation with height), and the lower the Benn parameter (Burton 2007, 2015). One might therefore have expected the Benn parameter in the general population to have declined over time, since the 1970s, alongside the development of the obesity epidemic (Flegal et al. 1998; Johnson et al. 2015). Alternatively, a secular increase in weight variation over time would have the opposite effect and increase the Benn parameter. How and why the Benn parameter has changed over time in response to shifts in the socio-political, behavioural, and nutritional landscape has not however previously been investigated.

Using longitudinal data from four large nationally-representative United Kingdom (UK) birth cohort studies, we aimed to investigate how the weight-height relationship, captured by the Benn parameter, changes across age (10–65 years), differs between sexes and varies according to time (1956–2015).

## Subject and methods

### Study samples

The 1946 Medical Research Council National Survey of Health and Development (1946 NSHD) is based on a sample (*N* = 5362) born in one week in March 1946 in England, Scotland, and Wales. The sample comprises all singleton births from women with husbands in non-manual and agricultural employment and a random selection of one in four singleton births to females with husbands in manual employment (Wadsworth et al. 2006; Kuh et al. 2011). The 1958 National Child Development Study (1958 NCDS) is based on 17,638 people born in one week in March 1958 in England, Scotland, and Wales; 920 immigrants born in the same week were incorporated during childhood (Power and Elliott 2006). A similar strategy was used in the 1970 British Cohort Study (1970 BCS), which is based on 17,287 people born in one week in April 1970, with the addition of 1814 individuals who were (1) born in Northern Ireland and included only in the birth sweep, (2) an immigrant who was incorporated into the study in childhood, or (3) never took part in any sweep (Elliott and Shepherd 2006). Finally, the 2001 Millennium Cohort Study (2001 MCS) is based on 18,818 people born between September 2000 and January 2002 who were living in England, Scotland, Wales, or Northern Ireland at age 9 months (Hansen 2012). All of the studies have received ethical approval and obtained informed parental and/or participant consent; this information is available from the study websites and/or cohort profiles.

For inclusion in the present study, participants were required to have at least one measurement of BMI during the studied age range. The resulting sample size in each study (1946 NSHD, *N* = 4724; 1958 NCDS, *N* = 16,307; 1970 BCS, *N* = 15,437; 2001 MCS, *N* = 13,249) represents more than 70% of the full cohort.

### Data

In the 1946 NSHD, weight and height were assessed at data collection sweeps at target ages of 11, 15, 20 (self-reported), 26 (self-reported), 36, 43, 53, and 60–64 years. In the 1958 NCDS, weight and height were assessed at data collection sweeps at target ages of 11, 16, 23 (self-reported), 33, 42 (self-reported), 44, and 50 (self-reported) years. In the 1970 BCS, weight and height were assessed at data collection sweeps at target ages of 10, 16 (one-third self-reported), 26 (self-reported), 30 (self-reported), 34 (self-reported), and 42 (self-reported) years. In the 2001 MCS, weight and height were assessed at data collection sweeps at target ages of 11 and 14 years. These data have been collated and cleaned as part of the Cohort and Longitudinal Studies Enhancement Resources (CLOSER) initiative (Johnson et al. 2015). The data and supporting metadata are available from the UK Data Archive.

In total, there were 26,168 observations of BMI in the 1946 NSHD, 74,975 in the 1958 NCDS, 56,275 in the 1970 BCS, and 23,279 in the 2001 MCS. In the 1946 NSHD, 79% of the sample had four or more serial observations and 68% had observations spanning more than 30 years. In the 1958 NCDS, 81% of the sample had three or more serial observations and 69% had observations spanning more than 25 years. In the 1970 BCS, 72% of the sample had three or more serial observations and 62% had observations spanning more than 20 years.

### Statistical analyses

BMI was computed as weight (kg)/height (m)^2^. Means and SDs for weight, height, and BMI at each sweep were produced stratified by sex and study. Correlations among height, weight, and BMI at each sweep were also produced stratified by sex and study.

The Benn parameter is the coefficient from the regression of log weight on log height (Benn 1971). Cross-sectional estimates were obtained by performing this regression for each study, sex, and sweep separately. All cohorts had a sweep at ages 10–11 years and three of the cohorts had a sweep at ages 42–43 years. Sex stratified regression models were applied to data pooled across the cohorts at each of the two ages. These models incorporated cohort × log height interaction terms to formally test for differences in the Benn parameter between cohorts.

Longitudinal analyses were then conducted in the 1946 NSHD, 1958 NCDS, and 1970 BCS to make use of the power of the serial data in these cohorts. For each sex and cohort separately, we modelled log weight against log height (centred at the mean) in a multilevel general linear regression framework (measurement occasion at level one and individuals at level two) with a random intercept (Equation (1) (Johnson et al. 2013a; Johnson 2015). Age was included as a fixed effect using a restricted cubic spline, with seven knots in the 1946 NSHD, six knots in the 1958 NCDS, and five knots in the 1970 BCS. Default knot locations were used based on Harrell’s (2001) recommended quantiles. The log height (or Benn) parameter was allowed to change over age through its interaction with the age spline terms. An adjustment was made for whether the data were measured or self-reported. Further, we allowed the level one variance (i.e. error) to differ according to whether the data were measured or self-reported. The model formula is given as

$${\ln\left(\mathrm{weight}\right)}_{ij}=\;\beta_{0j}+\;\beta_{1}{\ln(\mathrm{height})}_{ij}+\;\beta_{2-7}{\mathrm{Spline}}_{2-7ij}+\;\beta_{8-13}[{{\ln\left(\mathrm{height}\right)}_{ij}*\mathrm{Spline}}_{2-7ij}]\;+\;\beta_{14}{\mathrm{Selfreported}}_{ij}\;+\;\varepsilon_{1ij}{\mathrm{Selfreported}}_{ij}\;+\;\varepsilon_{2ij}{\mathrm{Measured}}_{ij}$$

$$\beta_{0j}=\;\beta_{0}+\;\mu_{0j}$$

$$\left[\mu_{0j}\right]\;∼N\left(0,{Ω}_{\mu }\right):\;{Ω}_{\mu }=\;\left[\sigma_{\mu 0}^{2}\right]$$

$$\left[\begin{array}{c} \varepsilon_{1ij}\\ \varepsilon_{2ij} \end{array}\right]\;∼N\left(0,{Ω}_{\varepsilon }\right):\;{Ω}_{\varepsilon }=\;\left[\begin{array}{cc} \sigma_{\varepsilon 1}^{2} & \\ 0 & \sigma_{\varepsilon 2}^{2} \end{array}\right]$$

where ${\ln\left(\mathrm{weight}\right)}_{ij}$ and ${ln(\mathrm{height})}_{ij}$ are the natural logged values at age i of person $j.\;{\mathrm{Spline}}_{2-7ij}$ is a restricted cubic spline of decimal age with up to seven knots. ${\mathrm{Selfreported}}_{ij}$ is a dummy variable contrasting self-reported data against measured data. $\beta_{0-14}$ are fixed effects. $\mu_{0j}\;is$ a random effect. $e_{1ij}$ is an error term for self-reported data and $e_{2ij}$ is an error term for measured data. The $\mu_{0j}\;$ is assumed to be normally distributed with mean zero and variance $\sigma_{\mu 0}^{2}.$ The $e_{1ij}$ and $e_{2ij}$ are assumed to follow normal distributions with zero means, variances $\sigma_{\varepsilon 1}^{2}$ and $\sigma_{\varepsilon 2}^{2},$ and covariance zero.

The value of the Benn parameter at age i is then calculated as

$${\mathrm{Benn}\;\mathrm{parameter}}_{i}\;=\;\beta_{1}+\;\beta_{8-13}{\mathrm{Spline}}_{2-7i}$$

The models were used to estimate mean trajectories describing how the Benn parameter changes over age. Trajectories for the three cohorts were plotted together in a single figure, for each sex, in order to visualise secular differences in the Benn parameter.

All procedures were performed in Stata 15 (StataCorp LP, College Station, TX, USA). The command runmlwin was used for the multilevel models (Leckie G and Charlton C 2012).

## Results

The mean values of weight, height, and BMI are shown in Table 1. Weight and BMI increased with age in each sex and study, while height was reassuringly stable across adulthood. Expected secular differences in BMI values were observed. For example, mean BMI at ages 10/11 years in males was 17.3 kg/m^2^ in the 1946 NSHD and 1958 NCDS, 16.7 kg/m^2^ in the 1970 BCS, and 19.1 kg/m^2^ in the 2001 MCS. Similarly, mean BMI at ages 42/43 years in males was 25.7 kg/m^2^ in the 1946 NSHD, 26.4 kg/m^2^ in the 1958 NCDS, and 27.5 kg/m^2^ in the 1970 BCS.

**Table 1.** Weight, height, and BMI means.

				Males				Females		
	Age	Date	*N*	Weight (kg)	Height (cm)	BMI (kg/m^2^)	*N*	Weight (kg)	Height (cm)	BMI (kg/m^2^)
1946 NSHD										
2461 males	11	1957	2050	34.3	140.6	17.3	1887	34.9	140.8	17.5
2263 females	15	1961	1881	51.8	162.0	19.6	1700	51.9	158.4	20.6
	20	1966	1802	70.9	176.9	22.6	1629	57.7	162.7	21.8
	26	1972	1822	73.6	177.1	23.4	1782	59.0	162.4	22.4
	36	1982	1631	76.5	175.3	24.8	1618	62.1	162.3	23.5
	43	1989	1612	79.0	175.3	25.7	1595	66.2	162.3	25.2
	53	1999	1451	83.7	174.6	27.4	1494	71.6	161.6	27.5
	60–64	2006–2010	1059	85.4	174.8	27.9	1155	72.9	161.7	27.9
1958 NCDS										
8357 males	11	1969	6381	36.0	143.9	17.3	6117	37.2	144.8	17.6
7950 females	16	1974	5697	58.9	170.2	20.2	5342	54.4	160.9	21.0
	23	1981	6124	72.7	177.3	23.1	6145	58.2	162.2	21.1
	33	1991	5379	79.8	176.8	25.5	5321	65.1	162.9	24.5
	42	2000	5465	83.3	177.3	26.4	5574	67.0	162.6	25.3
	44	2002	4576	86.3	175.9	27.8	4618	71.2	162.5	27.0
	50	2008	4139	87.0	176.2	28.0	4097	70.7	162.6	26.8
1970 BCS										
7923 males	10	1980	6251	32.2	138.7	16.7	5908	32.8	138.4	17.0
7514 females	16	1986	3713	63.1	173.8	20.8	4213	56.5	162.8	21.3
	26	1996	2576	74.9	175.3	24.4	4697	62.8	164.6	23.2
	30	2000	5313	81.4	178.5	25.5	5559	65.4	164.2	24.3
	34	2004	4521	84.6	178.5	26.5	4794	67.9	164.2	25.2
	42	2012	4298	87.7	178.6	27.5	4433	70.6	164.2	26.2
2001 MCS										
6702 males	11	2012	6342	40.9	145.9	19.1	6238	42.2	146.7	19.4
6547 females	14	2015	5406	58.6	166.8	21.0	5293	57.1	161.0	21.9

The SDs for weight showed a similar pattern of between-cohort differences (Table 2). For example, the weight SD at ages 10/11 years in males was 6.0 kg/m^2^ in the 1946 NSHD, 6.9 kg/m^2^ in the 1958 NCDS, 4.9 kg/m^2^ in the 1970 BCS, and 9.9 kg/m^2^ in the 2001 MCS. Similarly, the weight SD at ages 42/43 years in males was 12.1 kg/m^2^ in the 1946 NSHD, 13.7 kg/m^2^ in the 1958 NCDS, and 15.7 kg/m^2^ in the 1970 BCS. Because the height SDs were approximately the same in each cohort (at comparable ages), the BMI SDs demonstrated a similar pattern of between-cohort differences as those for weight.

**Table 2.** Weight, height, and BMI standard deviations.

			Males			Females		
	Age	Date	Weight (kg)	Height (cm)	BMI (kg/m^2^)	Weight (kg)	Height (cm)	BMI (kg/m^2^)
1946 NSHD								
	11	1957	6.0	6.7	2.1	7.1	7.1	2.6
	15	1961	9.6	8.9	2.4	8.8	6.3	3.0
	20	1966	9.2	6.8	2.5	8.4	6.3	2.9
	26	1972	10.0	6.5	2.8	9.2	6.4	3.2
	36	1982	11.4	6.6	3.2	11.3	6.1	4.1
	43	1989	12.1	6.6	3.5	12.8	6.0	4.8
	53	1999	13.6	6.6	4.0	14.3	6.0	5.5
	60–64	2006–2010	13.5	6.6	4.1	14.1	5.9	5.3
1958 NCDS								
	11	1969	6.9	6.9	2.4	7.7	7.5	2.7
	16	1974	10.3	7.9	2.7	8.5	6.2	3.0
	23	1981	10.4	7.0	2.9	9.1	6.6	3.2
	33	1991	12.9	6.7	3.7	13.4	6.4	4.9
	42	2000	13.7	6.7	3.9	13.6	6.6	5.0
	44	2002	14.6	6.6	4.3	15.2	6.2	5.6
	50	2008	15.3	6.6	4.5	15.0	6.2	5.5
1970 BCS								
	10	1980	4.9	6.2	1.9	5.7	6.4	2.3
	16	1986	10.4	8.5	3.0	8.8	6.7	3.1
	26	1996	11.1	5.2	3.4	11.2	6.8	4.0
	30	2000	13.5	7.2	3.8	13.0	6.9	4.7
	34	2004	14.6	7.2	4.2	14.1	6.8	5.1
	42	2012	15.7	7.1	4.6	15.3	6.9	5.6
2001 MCS								
	11	2012	9.9	7.1	3.6	10.3	7.4	3.7
	14	2015	13.6	8.7	4.0	12.0	6.4	4.2

As shown in Table 3, the weight–height correlations generally decreased over age and were lower in females than males. They also appeared to decrease over time, most noticeably in adulthood in males. For example, the correlation at ages 42/43 years in males was 0.46 in the 1946 NSHD, 0.44 in the 1958 NCDS, and 0.39 in the 1970 BCS. The BMI-height correlations were positive at age 10/11 years but switched to be negative at age 20 years in the 1946 NSHD, age 23 years for males and age 16 years for females in the 1958 NCDS, and age 16 years in the 1970 BCS. Further, the negative adulthood BMI-height correlations were consistently stronger for females than males.

**Table 3.** Weight, height, and BMI correlations.

			Males			Females		
	Age	Date	Weight Height	BMI Weight	BMI Height	Weight Height	BMI Weight	BMI Height
1946 NSHD								
	11	1957	0.69	0.84	0.19	0.66	0.87	0.22
	15	1961	0.75	0.82	0.26	0.50	0.88	0.04
	20	1966	0.55	0.81	−0.05	0.45	0.84	−0.10
	26	1972	0.48	0.84	−0.07	0.39	0.87	−0.12
	36	1982	0.49	0.86	−0.02	0.34	0.91	−0.08
	43	1989	0.46	0.87	−0.03	0.26	0.92	−0.13
	53	1999	0.42	0.89	−0.04	0.22	0.93	−0.16
	60–64	2006–2010	0.39	0.88	−0.09	0.26	0.92	−0.12
1958 NCDS								
	11	1969	0.68	0.87	0.23	0.68	0.87	0.24
	16	1974	0.65	0.86	0.17	0.44	0.87	−0.06
	23	1981	0.49	0.83	−0.07	0.39	0.85	−0.14
	33	1991	0.43	0.88	−0.04	0.29	0.92	−0.10
	42	2000	0.44	0.88	−0.02	0.29	0.91	−0.12
	44	2002	0.41	0.89	−0.04	0.27	0.93	−0.09
	50	2008	0.40	0.90	−0.03	0.27	0.93	−0.10
1970 BCS								
	10	1980	0.65	0.81	0.08	0.64	0.84	0.13
	16	1986	0.54	0.80	−0.07	0.40	0.84	−0.15
	26	1996	0.34	0.91	−0.07	0.33	0.89	−0.13
	30	2000	0.44	0.87	−0.05	0.29	0.90	−0.14
	34	2004	0.42	0.88	−0.05	0.26	0.91	−0.14
	42	2012	0.39	0.89	−0.06	0.24	0.92	−0.15
2001 MCS								
	11	2012	0.64	0.92	0.30	0.65	0.92	0.30
	14	2015	0.57	0.90	0.16	0.43	0.93	0.07

Cross-sectional estimates of the Benn parameter are presented in Table 4. At ages 10/11 years, the Benn parameter was greater than 3 in both sexes in the 2001 MCS but between 2.2 and 2.7 in the three older cohorts. Compared to the 1946 NSHD, this cohort difference was estimated to be +0.67 (0.53, 0.81) in males and +0.53 (0.38, 0.68) in females (Table 5). Conversely, at ages 42/43 years, the Benn parameter was lowest in the 1970 BCS. Compared to the 1946 NSHD, this cohort difference was, however, estimated to only be −0.12 (−0.34, 0.10) in males and −0.15 (−0.42, 0.13) in females.

**Table 4.** Cross-sectional estimates of the Benn parameter.

	Age	Date	Males	Females
1946 NSHD				
	11	1957	2.44	2.58
	15	1961	2.60	2.12
	20	1966	1.87	1.70
	26	1972	1.77	1.56
	36	1982	1.95	1.67
	43	1989	1.90	1.39
	53	1999	1.82	1.21
	60–64	2006–2010	1.66	1.41
1958 NCDS				
	11	1969	2.61	2.69
	16	1974	2.50	1.79
	23	1981	1.79	1.51
	33	1991	1.85	1.52
	42	2000	1.92	1.46
	44	2002	1.84	1.52
	50	2008	1.87	1.49
1970 BCS				
	10	1980	2.20	2.36
	16	1986	1.86	1.51
	26	1996	1.71	1.48
	30	2000	1.84	1.39
	34	2004	1.81	1.32
	42	2012	1.78	1.24
2001 MCS				
	11	2012	3.11	3.12
	14	2015	2.61	2.34

**Table 5.** Estimated differences in the Benn parameter between cohorts at two ages.

	Males			Females		
	*B*	95% CI	*p*	*B*	95% CI	*p*
10/11 years						
1946 NSHD (referent)	–	–	–	–	–	–
1958 NCDS	0.17	0.03, 0.31	.02	0.10	−0.05, 0.25	.18
1970 BCS	−0.24	−0.38, −0.10	.001	−0.22	−0.38, −0.07	.005
2001 MCS	0.67	0.53, 0.81	<.001	0.53	0.38, 0.68	<.001
42/43 years						
1946 NSHD (referent)	–	–	–	–	–	–
1958 NCDS	0.02	−0.20, 0.23	.88	0.08	−0.19, 0.35	.57
1970 BCS	−0.12	−0.34, 0.10	.28	−0.15	−0.42, 0.13	.30

**Figure 1.**
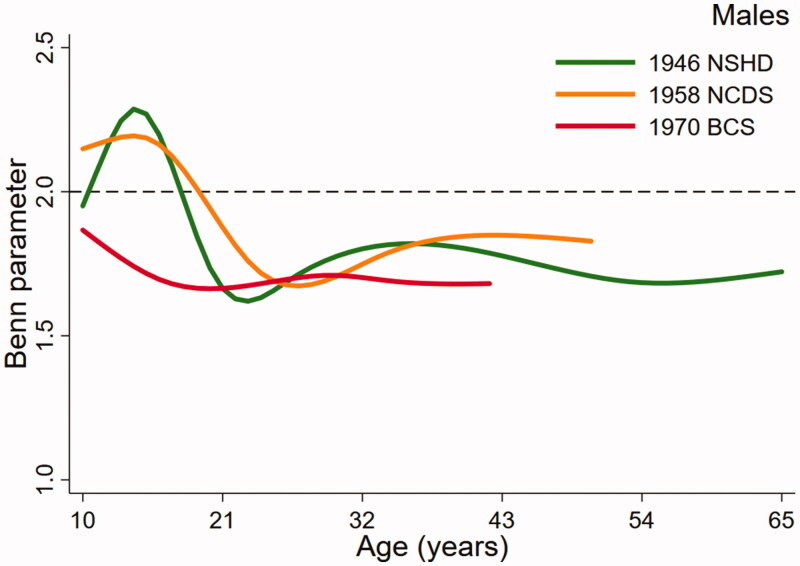
Benn parameter trajectories for males, estimated using multilevel models.

Figures 1 (males) and 2 (females), the key output from the multilevel models, present trajectories describing how the Benn parameter changes over age in study. The dashed reference line at 2 represents the height power for BMI (i.e. kg/m^2^). The Benn parameter for each sex and cohort was closest to 2 in childhood but was consistently lower than 2 across adulthood, particularly in females. For example, the estimated Benn parameter for females in the 1970 BCS was close to 1 at 42 years of age. There were also noticeable differences in the trajectories between the studies. Males in the 1946 NSHD demonstrated a clear peak in the Benn parameter in adolescence that was less obvious in the 1958 NCDS and not present at all in the 1970 BCS. For females, the shape of the trajectory was similar in each cohort, declining across adolescence before plateauing in adulthood. In both sexes, the trajectory for the 1970 cohort was generally lower than the trajectories for the 1946 NSHD and 1958 NCDS cohorts. A secular trend towards a lower Benn parameter trajectory was, however, clearer in females than males.

**Figure 2.**
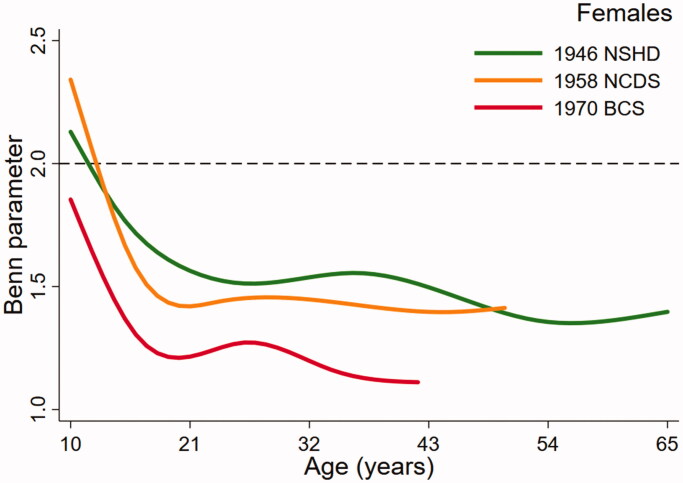
Benn parameter trajectories for females, estimated using multilevel models.

## Discussion

In this paper, we demonstrate how the best power with which to raise height, termed the Benn parameter, changes over age and is lower in females compared to males. The key and novel findings, however, are that the Benn parameter was slightly lower in the 1970 cohort compared to the older cohorts because of a slightly lower weight-height correlation, but much higher in childhood in the 2001 cohort compared to the older cohorts because of much greater variation in weight.

Previous studies have derived age- and sex-specific height scaling powers for the computation of the Benn index. Cole (1986) reported that the optimal power is approximately 2 in pre-school children, increasing to a peak of around 3 in puberty that occurred 18-months earlier in girls than in boys, before decreasing into adulthood. The fact that we only observed a peak in adolescence in males in the 1946 NSHD is probably because we only had childhood/adolescent data at 10/11 and 15/16 years of age, so had no information to identify a peak in between these ages. Peterson et al (2017) similarly found limited evidence of a peak in a recent analysis of the 1999–2006 US National Health and Nutrition Examination Surveys (NHANES), perhaps owing to the data being cross-sectional and grouped into age bins (e.g. 12–13 years). Other studies have similarly investigated the Benn parameter at various ages and in different populations; values during adulthood are normally between 1.1 and 2.5 and typically lower in women than in men (Goldbourt and Medalie 1974; Lee et al. 1981; Garn and Pesick 1982; Micozzi et al. 1986; Revicki and Israel 1986; Nevill and Holder 1995). No studies have, however, modelled longitudinal data to produce trajectories of the Benn parameter spanning up to 55 years of life, as we have done in the present paper. Sperrin et al (2016) have produced trajectories based on serial cross-sectional Health Survey for England data and observed the Benn parameter to be fairly stable and approximate 2.0 for men and 1.5 for women between 20 and 70 years of age. They did not find any evidence that the Benn parameter had changed over time between 1991 and 2011 but did conclude that ‘longitudinal studies across a variety of cohorts samples’ are needed (Sperrin et al. 2016).

The proportion of a person’s weight that is fat, as opposed to lean tissue or bone, is on average higher among women than men (Heo et al. 2012). Fat has a lower correlation with height than muscle does (Burton 2007, 2015) resulting in a lower weight–height correlation in females compared to males, as documented in the present study. A lower weight–height correlation results in a lower Benn parameter, so it makes sense that height needs to be raised to a lower power for females compared to males. Fat is also more variable than fat-free mass (Burton 2007), which has an opposing effect and increases the Benn parameter. However, at least as far as sex differences are concerned, it appears that this opposing effect does not overpower the effect of the lower weight–height correlation.

Different levels of variation in, and correlation between, weight and height can help explain why the Benn parameter takes different values in different groups of individuals, be these morphological, geographical, or secular. Here, we demonstrate that the Benn parameter is not static over time but has altered in response to shifts in the socio-political, behavioural, and nutritional landscape. The obesity epidemic really started in the 1970s (Flegal et al. 1998; Johnson et al. 2012a; Johnson et al. 2015) and the 1970 BCS cohort has therefore experienced the worsening obesogenic environment across their entire lives. The Benn parameter tended to be slightly lower in the 1970 BCS compared to the older cohorts, because of a slightly lower weight–height correlation. This secular difference was clearer in females than males; this could be explained by a stronger positive secular trend in adiposity in females than males, although evidence to support this theory is mixed (Okosun et al. 2004; Li et al. 2006; Li et al. 2007; Johnson et al. 2013b; Freedman et al. 2017). The estimated difference in the Benn parameter between the 1970 BCS and the 1946 NSHD was larger at ages 10/11 years than 42/43 years, perhaps because at later ages, the environment was more obesogenic and this increased the weight SD (which has the opposite effect on the Benn parameter compared to the decreasing weight-height correlation). It, therefore, follows that the Benn parameter was much higher in childhood in the 2001 cohort compared to the older cohorts because of much greater variation in weight but only a marginally lower weight-height correlation. The finding that the Benn parameter was approximately 3 in contemporary pubertal children is in agreement with literature reporting the tri-ponderal mass index (kg/m^3^) to be a better indicator of body fat levels in modern-day adolescences than BMI (Peterson et al. 2017).

Despite the BMI not being computed using the power that best minimises its correlation with height, the index is ubiquitous in obesity research and practice. A different height scaling power has not been universally accepted and applied, perhaps because changing the Benn parameter apparently has little influence on the resulting index. For example, in males at age 60–64 years in the 1946 NSHD, the correlation between kg/m^2^ and kg/m^1.5^ is 0.99 and the percentage variation in each index explained by height is 0.4% and 0.07%, respectively. Such a high correlation does not, however, mean that BMI is not a biased weight-for-height index. Using the example above, the concordance (which incorporates a bias correction factor) is actually only 0.45 (Lin 1989). Even if there are no biases, the idea that the BMI should ideally be uncorrelated with height is questionable. As shown by Benn (1971), minimising the correlation of a weight-for-height index with height only maximises the association between the index and adiposity if height and adiposity are uncorrelated. This is not normally true, particularly during childhood and adolescence when there are strong temporal relationships between skeletal dimensions and tissue accretion during growth and development (Dunger et al. 2005; Johnson et al. 2012b; Cole et al. 2014; Bell et al. 2018). Garn and Haskell (1959, 1960) showed that children with greater levels of body fat were indeed taller and entered puberty earlier, and this finding has been replicated more recently in the EarlyBird study (Metcalf et al. 2011). This might explain why we observed small positive BMI–height correlations in childhood/adolescence in the present study. If the purpose of a weight-for-height index is to assess health risk, rather than weight independent of height *per se*, then making the index uncorrelated with height might not be the goal. Indeed, being tall in childhood/adolescence or short in adulthood are associated with various non-communicable diseases, independently of adiposity (Emerging Risk Factors Collaboration 2012; Grijalva-Eternod et al. 2013; Wells and Cole 2014). The idea that part of the risk apportioned by BMI is not due to adiposity is not often thoroughly considered but could be tested empirically in epidemiology papers (Wells 2014; Gracia-Marco et al. 2016).

### An evolutionary perspective

Evolutionary life history theory assumes that the resources available to an organism in any environment are finite, and that each organism has been selected to allocate those resources in ways that maximise reproductive fitness (Stearns 1992). The four key functions are maintenance, growth, reproduction, and defence (Wells et al. 2017). Height is clearly a marker of growth, either ongoing or completed. Fat-free mass is a marker not only of growth but also of maintenance (given that it incorporates the vital organs) and reproduction (since muscle mass promotes sexual signalling in males, while fat-free mass is a stronger predictor of offspring birth weight in females). Fat mass can be considered a marker of defence, as adipose tissue contributes both energy and molecular precursors to immune function (Wells et al. 2019). Trade-offs between different life history functions may manifest at the level of these body components and this may explain why associations between height, fat mass, and fat-free mass may vary substantially between population sub-groups. In Peru, for example, the correlation between childhood height and waist circumference is negative in harsher high-altitude settings (indicating a trade-off between growth and defence) but positive in more favourable lowland settings (Pomeroy et al. 2014). These insights may help understand the variable values taken by the Benn index observed in the present study, where the emergence of the obesogenic niche is altering ways in which height growth is correlated with accretion of fat mass and fat-free mass. If both the numerator (the sum of fat mass and fat-free mass) and the denominator (height) not only respond similarly to ecological factors but also do so in coordinated ways that vary by age and setting, then a single statistical solution may be unrealistic.

### Strengths and limitations

The key strength of this paper lies in the longitudinal analysis on the relationship of weight with height across age (10–65 years) and time (1956–2015) in 49,717 males and females with 180,697 serial observations. Attrition over follow-up was observed (e.g. 43% fewer observations at age 60–64 years compared to age 11 years in the 1946 NSHD), but this is handled by the multilevel models under a missing at random assumption. While the four birth cohort studies were designed to be nationally representative at initiation, ethnic diversity is low and even in the 1970 BCS sample only about 5% were non-white British. In the 2001 MCS sample approximately 15% were non-white British. The association of BMI with body fat differs between ethnicities (Hudda et al. 2017, 2018), but the extent to which this is because the weight-height relationship is not properly captured by raising height to a power of 2 in some ethic groups needs further investigation. Body composition data are limited in the studies, so we were not able to assess the relationship of BMI with body composition (over age, time, and sex) and how this might depend on the Benn parameter.

## Conclusion

This paper demonstrates how the Benn parameter (the optimal height scaling power to create an index of weight that is minimally correlated with height) is not consistent because the degree to which weight and height vary and correlate with each other differs between sexes and changes across age and time. Changes over time in the obesogenic environment appear to have first reduced the Benn parameter due to a lowering of the weight–height correlation but second and more drastically increased the Benn parameter due to increasing weight variation. If population levels of obesity continue to rise as a result of the weight distribution becoming increasingly right skewed, the call to replace BMI with the tri-ponderal mass index (kg/m^3^) may become more valid.
